# Data on Field Canals Improvement Projects for Cost Prediction Using Artificial Intelligence

**DOI:** 10.1016/j.dib.2020.105688

**Published:** 2020-05-19

**Authors:** Haytham H. Elmousalami

**Affiliations:** a Project management professional (PMP) at general petroleum company (GPC), Egypt; b Researcher at Faculty of Computers and Artificial Intelligence, Cairo University, Egypt

**Keywords:** Artificial Intelligence, Ensemble machine learning, Delphi rounds, Questionnaire survey, Conceptual cost, Conceptual duration, Project management

## Abstract

Field Canals Improvement Projects is an important sustainable project to save fresh water in our world. Machine learning and artificial intelligence (AI) needs sufficient dataset size to model and predict the cost and duration of Field Canals Improvement Projects. Therefore, this data paper presents dataset includes the key parameters of such project to be used for analyzing and modelling project cost and duration. The data were acquired based on questionnaire survey and collecting historical cases of Field Canals Improvement Projects. The data consists of the following features: area served, total length of PVC pipe line, number of irrigation values, construction year, geographical zone, cost of FCIP, and duration of FCIP construction. The data can be applied to compare and evaluate the performance of machine learning algorithms for predicting cost and duration.

**Specifications Table**

**Table:** 

Subject	Management Information Systems.
Specific subject area	Predictive analysis, conceptual cost estimate, duration prediction, algorithms validation, ensemble machine learning.
Type of data	Table, Excel file (7 columns X 1276 rows).
How data were acquired	The data were acquired based on questionnaire survey and collecting historical cases of Field Canals Improvement Projects as shown in Table 1 and Appendix B. Moreover, Delphi rounds [9] have been conducted as displayed in Appendix C.
Data format	Raw data.
Parameters for data collection	The data consists of the following features: area served, total length of PVC pipe line, number of irrigation values, construction year, geographical zone, cost of FCIP, and duration of FCIP construction.
Description of data collection	Key conceptual cost drivers affecting the cost estimation of FCIPs based on using historical quantitative data. Raw data are publicity available on the following repository.
Data source location	Delta region in Egypt.
Data accessibility	Data are within this article.
Related research article	Author's name: Haytham H. Elmousalami Title: Artificial Intelligence and Parametric Construction Cost Estimate Modeling: State-of-the-Art Review Journal: Journal of Construction Engineering and Management, Volume 146 Issue 1 - January 2020 DOI: https://doi.org/10.1061/(ASCE)CO.1943-7862.0001678

## Value of the data

• The data explains the key cost drivers of Field Canals Improvement Projects (FCIPs).
• The dataset is important for irrigation authorities and stakeholders such as contractors, engineers and decision makers to estimate the conceptual cost of FCIPs based on financial and feasibility perspectives.
• The data objective is developing a reliable parametric cost or duration estimation model at the conceptual phase for Field Canals Improvement Projects (FCIPs).
• The data can be applied to compare and evaluate the performance of machine learning algorithms for predicting cost and duration.
• Data can be used as a benchmark data to assess the accuracy of other novel frameworks or models against the developed models in the previous studies [1].
• The data can be conducted to applied advanced computational theories and algorithms such as fuzzy-genetic model and deep learning algorithms.

## 1 Data Description

**Fig. 1:**
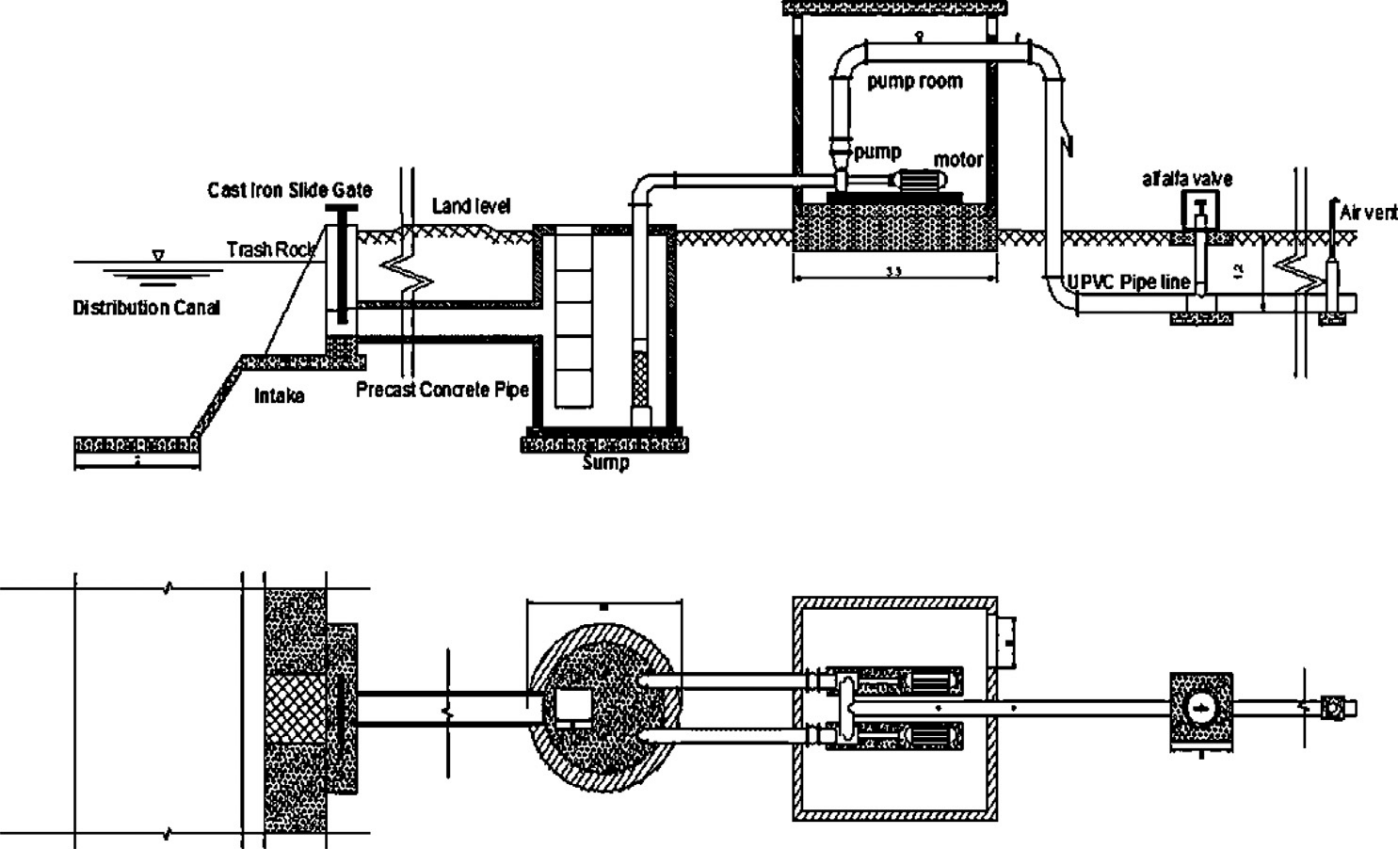
The general layout for buried PVC pipelines Mesqa (lateral canal).

**Fig. 2:**
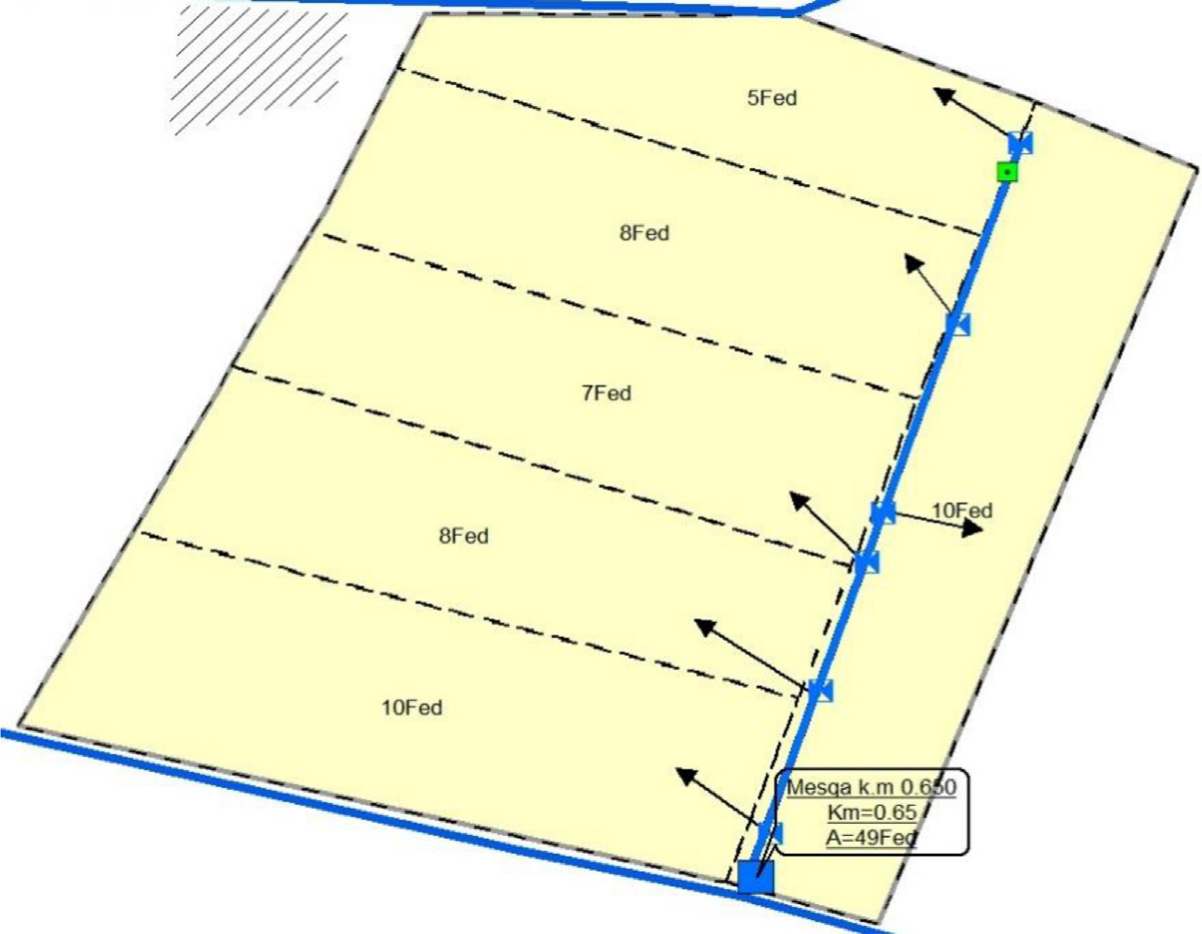
GIS picture for FCIP planning at 0.65 km on Soltani Canal.

Construction cost estimation can be applied for several projects such as Irrigation [1], transportation [2,5], petroleum exploration and safety [4,6]. The current trend of cost estimation is using Artificial intelligence and machine learning to get the most accurate cost predictions [3]. Artificial intelligence and machine learning require sufficient dataset to model the cost prediction. The objective of this paper is describing the data on FCIPs for conceptual cost prediction using artificial intelligence and data science. The data are presenting the key conceptual cost drivers affecting the cost estimation of FCIPs based on using historical quantitative data. The collected parameters are denoted from P1 to D where minimum (Min), maximum (Max) and standard deviation (StD) have been displayed as showed in Table 1. Such parameters are gathered based on surveying historical cases of Field Canals Improvement Projects via construction site records and contact information as quantitative data based on past project construction contracts’ information and site recordings from 2011 to 2018 [1, 7]. Such information is described in Table 1 and Appendix B. The geographical zone parameter is divided into three categories: 0 is the middle of the delta region, 1 is the east of delta, 2 is the west of delta. The poly venial chloride (PVC) pipeline diameters are ranging from 225 mm to 350 mm as shown in Fig. 1, Fig. 2. Moreover, these collected parameters depend on the previous literature.

**Table 1:** Descriptive statistics for the selected key project parameters.

Notation	parameter name	Unit	Min	Max	StD
P1	Area served	Hectare	19	106	19.166
P2	total length of PVC pipe line	meter	119	2075.45	406.3
P3	number of irrigation values	number	1	28.89	3.7543
P4	Construction year	year	2011	2018	1.4295
P5	Geographical zone	Zone	0	2	0.8058
C	Cost of FCIP	LE / FCIP	570000	3700000	884972
D	Duration of FCIP construction	day	58	133.525	11.975

## 2 Experimental design, materials, and methods

Questionnaire survey and collecting historical cases of Field Canals Improvement Projects have been conducted to collect the data using Delphi rounds as Appendix C. The Delphi rounds consists of three main rounds: collecting, rating and revising rounds [9]. The collecting round collects the all possible parameters. Rating and revising rounds have been applied to assessing and ranking the parameters. Accordingly, the key parameters were the top-rated parameters based on expert's evaluation. This approach was the identifying the key parameters based on qualitative technique.

**Fig. 3:**
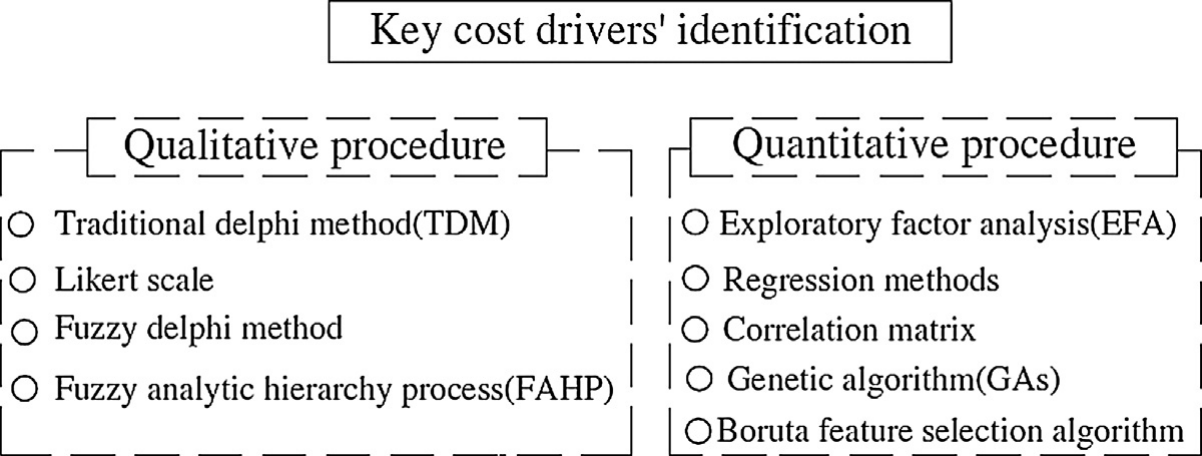
Qualitative and quantitative procedure.

Developing a reliable parametric cost estimation model consists of two main stages: key cost drivers identification and machine learning model development [7,10]. Firstly, identifying the key conceptual drivers can be conducted using qualitative or quantitative approaches as shown in Fig. 3. Selecting the key drivers are affecting the accuracy of the cost estimation of FCIPs is based on using historical quantitative data. The data objective aims identifying FCIPs’ cost drivers of preliminary cost estimate (CDPCE) by using the historical quantitative data. Experts’ opinions are not utilized here to avoid biased selection when using human judgment. The purpose of the data is to discover and apply data-driven methods to select the key cost drivers based only on the quantitative collected past data. The importance of cost drivers is to help decision makers to predict the preliminary cost of FCIPs and study the financial feasibility of these projects [1,7].

**Fig. 4:**
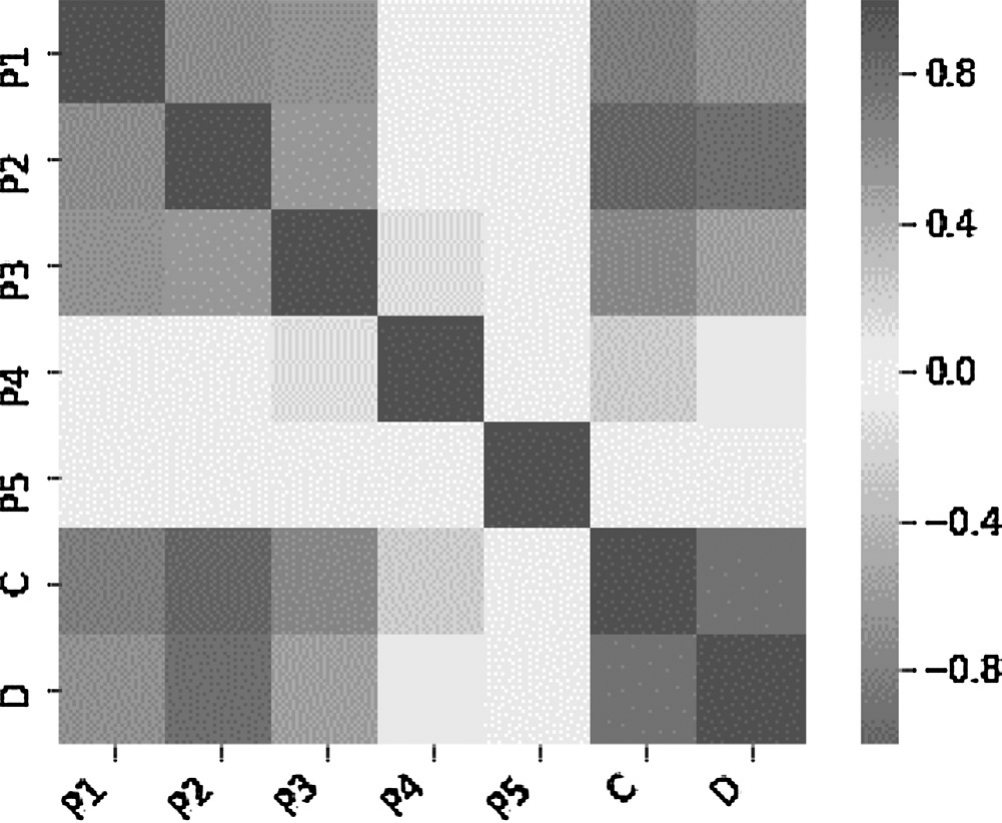
Heat map correlation for key parameters.

As shown in Fig. 4, the heat map correlation presents high positive correlation among the total length of PVC, cost of FCIP and the duration of FCIP. A slight positive correlation exists among the area served and the number of irrigation valves and duration of the project [1]. No strong correlation exists among the geographical zone of the project and project duration. Accordingly, this parameter can be removed. A slight negative correlation exists among the construction year and project duration. Accordingly, the heat map of key parameters correlation shows the pair relation among all the selected parameters [7].

**Fig. 5:**
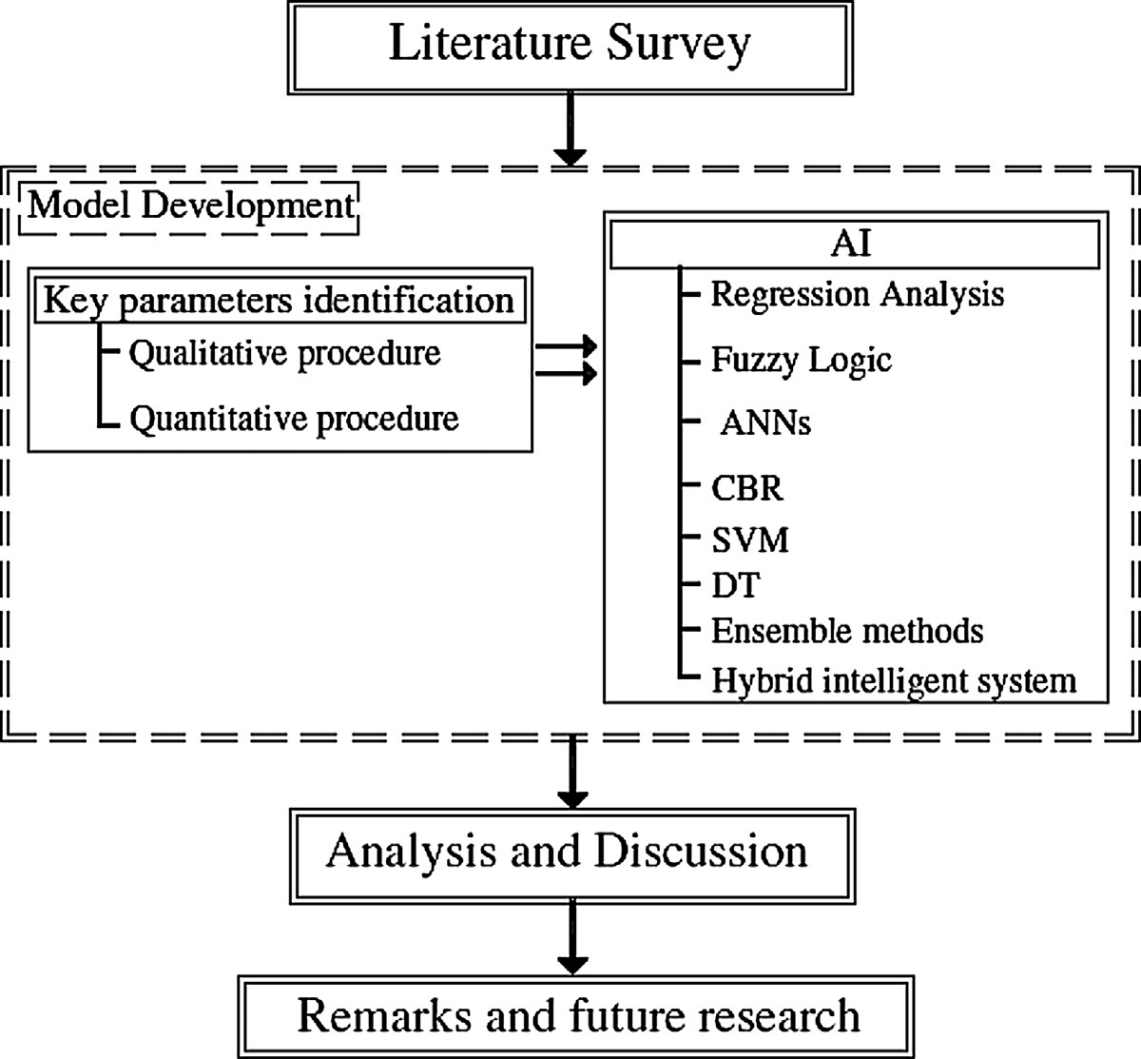
Research methodology.

Secondly, a comprehensive tool for parametric cost or duration estimation can be developed using ML algorithms such as multiple regression analysis and the optimum neural network model. The data objective is developing a reliable parametric cost estimation model before the construction of FCIPs. Therefore, a total of 1276 FCIPs of constructed projects are collected to build up the proposed model. This data can be used for executing the most common artificial intelligence (AI) techniques which are conducted for cost modeling such as fuzzy logic (FL) model, artificial neural networks (ANNs), regression model, case-based reasoning (CBR), hybrid models, and evolutionary computing (EC) such as genetic algorithm (GA)[1] as showed in Fig. 5.

## Figures and Tables

**Table:** 

Notation	parameter name	Unit	Min	Max	StD
P1	Area served	Hectare	19	106	19.166
P2	total length of PVC pipe line	meter	119	2075.45	406.3
P3	number of irrigation values	number	1	28.89	3.7543
P4	Construction year	year	2011	2018	1.4295
P5	Geographical zone	Zone	0	2	0.8058
C	Cost of FCIP	LE / FCIP	570000	3700000	884972
D	Duration of FCIP construction	day	58	133.525	11.975

**Table:** 

			**Degree of Importance**					**Notes**
**ID**	**Parameters categories**	**Parameters**	**1**	**2**	**3**	**4**	**5**	
P1	Civil	Area served (hectare)						
P2	Civil	Total length of PVC pipe line						
P3	Civil	Construction year						
P4	Civil	Mesqa discharge (capacity)						
P5	Mechanical	Number of Irrigation Valves (alfa-alfa valve)						
P6	Civil	Consultant performance and errors in design						
P7	Electrical	Number of electrical pumps						
P8	Civil	PVC pipe diameter						
P9	Location	Orientation of mesqa (intersecting with drains or roads or both)						
P10	Mechanical	Electrical and diesel pumps discharge						
P11	Civil	PC Intake, steel gate and Pitching with cement mortar						
P12	Location	Type of mesqa (Parallel to branch canal (Gannabya), Perpendicular on branch canal)						
P13	miscellaneous	Farmers Objections						
P14	Electrical	Electrical consumption board type						
P15	Location	Geographical zone						
P16	Civil	Pump house size 3m*3m or 3m*4m						
P17	miscellaneous	cement price						
P18	Mechanical	Head of electrical and diesel pumps						
P19	miscellaneous	Farmers adjustments						
P20	Civil	Sand filling						
P21	Civil	Sump size						
P22	Civil	Contractor performance and bad construction works						
P23	miscellaneous	pump price						
P24	Civil	Crops on submerged soils (Rice) and its season (May to July)						
P25	miscellaneous	pipe price						
P26	Location	Topography and land levels of command area						
P27	Civil	Construction planned durations						
P28	Civil	Pumping and suction pipes						
P29	Mechanical	Steel mechanical connections						
P30	Civil	Difference between land and water levels						
P31	miscellaneous	steel price						
P32	Civil	Number of PVC branches						
P33	miscellaneous	Cash for damaged crops						
P34	Mechanical	Air / Pressure relief valve						
P35	miscellaneous	Crops on unsubmerged soils (wheat, corn, cotton, etc.)						
C	Output	Cost of FCIP						
D	Output	Duration of FCIP construction						

**Table:** 

	**Respondents (Ri)**																
**ID**	**R1**	**R2**	**R3**	**R4**	**R5**	**R6**	**R7**	**R8**	**R9**	**R10**	**R11**	**R12**	**R13**	**R14**	**R15**	**Mean**	**SE**
P1	5	5	5	5	5	5	4	5	5	5	5	5	5	5	5	4.97	0
P2	4	4	4	4	4	5	4	4	5	5	5	4	5	5	5	4.43	0.1
P3	5	5	4	4	4	5	5	5	5	5	5	3	4	3	2	4.33	0.2
P4	4	5	4	5	4	2	4	4	5	4	5	5	4	3	4	4.33	0.2
P5	4	5	5	5	5	5	4	5	2	3	3	5	4	4	2	4.2	0.2
P6	5	5	5	5	5	4	3	4	4	4	5	4	3	4	2	4.13	0.2
	5	5	5	5	5	4	3	4	4	4	5	4	3	4	2	4.1	0.2
P8	5	5	5	5	5	2	2	4	5	5	4	4	5	1	4	3.9	0.2
P9	4	4	3	4	3	4	2	4	5	3	5	4	5	4	4	3.8	0.2
P10	5	3	4	5	5	1	4	3	4	4	4	2	4	5	5	3.57	0.1
P11	5	4	4	4	4	3	2	4	4	3	5	4	3	3	4	3.4	0.1
P12	5	5	3	2	3	1	4	3	4	4	4	1	4	4	5	3.07	0.2
P13	5	5	3	2	3	1	4	3	4	4	4	1	4	4	5	2.93	0.2
P14	4	3	3	4	4	4	2	4	4	3	4	3	2	4	4	2.87	0.2
P15	3	4	4	4	4	3	2	4	4	1	3	4	3	1	1	2.63	0.2
P16	1	3	3	1	1	4	2	1	3	5	4	5	4	5	2	2.6	0.2
P17	5	4	4	5	4	3	3	2	2	1	3	1	1	4	2	2.53	0.2
P18	4	4	4	5	3	1	2	3	3	4	3	3	2	2	2	2.53	0.2
P19	2	4	2	4	4	1	2	5	3	3	2	3	2	2	2	2.5	0.2
P20	3	4	2	2	3	2	4	1	3	1	2	2	3	4	4	2.4	0.2
P21	3	4	2	2	3	4	2	4	2	4	2	1	2	2	2	2.37	0.2
P22	2	2	2	2	3	3	2	4	2	5	2	5	2	1	1	2.2	0.2
P23	2	3	4	3	3	4	2	4	2	1	2	2	2	2	2	2.13	0.2
P24	2	2	2	2	2	3	4	4	2	4	2	4	2	1	2	2.1	0.2
P25	2	2	2	5	2	2	2	3	2	2	2	4	2	4	2	2.1	0.2
P26	2	3	3	3	5	1	2	3	2	2	2	2	2	2	2	2.1	0.1
P27	3	3	2	3	3	1	2	4	2	4	2	1	2	2	1	2.07	0.2
P28	1	3	4	3	4	3	1	3	1	2	1	2	1	5	1	2.07	0.2
P29	4	1	3	2	2	1	2	4	1	1	2	5	3	1	1	2	0.2
P30	2	1	1	1	3	4	3	1	3	4	3	2	2	1	2	1.9	0.1
P31	4	1	1	1	2	1	2	4	2	1	2	4	4	1	1	1.8	0.2
P32	2	3	1	2	4	1	2	1	2	3	4	1	2	1	2	1.8	0.2
P33	2	2	2	2	1	1	3	4	1	3	3	1	2	1	2	1.73	0.1
P34	4	2	2	2	1	2	1	2	1	4	1	1	2	1	2	1.6	0.2
P35	2	1	1	1	4	2	1	3	2	2	1	2	2	1	2	1.5	0.1
C	5	5	5	5	5	5	4	5	5	5	5	5	5	5	5	4.96	0
D	4	4	4	4	4	5	4	4	5	5	5	4	5	5	5	4.44	0.1

**Fig. C1:**
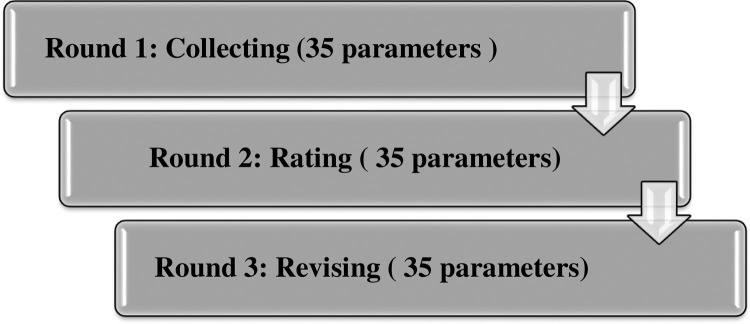
Delphi rounds.

## Appendix A. **Supplementary material**

Supplementary data associated with this article will be found in Excel (.csv) format in the online version and in the following public repository: https://github.com/HaythamElmousalami/Data_in-Breif.

## Appendix B: **Field survey module and contract information**

Likert scale is a rating scale to represent the opinions of experts where Likert scale can be consisted of three points, five points or seven points. For example, a five-point Likert scale may be “Extremely Important”, “Important”, “Moderately Important”, “Unimportant”, and “Extremely Unimportant” where the experts will select these points to answer received questions [4, 3, 8]. Based on Likert scale (5 points), select the most appropriate rate for each of the following parameters to evaluate each parameter affecting on the cost of FCIPs.

## Appendix C: **Delphi Rounds**

A Delphi rounds and Likert scale were used to determine the most important factors from viewpoints of consultant engineers and involved contractors sing three rounds: collecting parameter round, rating parameters round, and revising parameters round as shown in Fig. Appendix C Fig. C1 [8].

2. No Any relevant patents or copyrights exist.

## Appendix D Supplementary materials

Image, application 1
